# The Recent Technologies to Curb the Second-Wave of COVID-19 Pandemic

**DOI:** 10.1109/ACCESS.2021.3094400

**Published:** 2021-07-02

**Authors:** M. Poongodi, Mohit Malviya, Mounir Hamdi, Hafiz Tayyab Rauf, Seifedine Kadry, Orawit Thinnukool

**Affiliations:** College of Science and EngineeringHamad Bin Khalifa University, Qatar Foundation 370593 Doha Qatar; Department of CTO 5GWipro Ltd. 113418 Bengaluru 560035 India; Centre for Smart SystemsAI and Cybersecurity, Staffordshire University 7703 Stoke-on-Trent ST4 2DE U.K.; Faculty of Applied Computing and TechnologyNoroff University College 4608 Kristiansand Norway; Research Group of Embedded Systems and Mobile Application in Health Science, College of Arts, Media and TechnologyChiang Mai University 26682 Chiang Mai 50200 Thailand

**Keywords:** Epidemic, coronavirus, cloud, 5G, drone, X-Ray, CT-scan, telemedicine, artificial intelligence

## Abstract

Different epidemics, specially Coronavirus, have caused critical misfortunes in various fields like monetary deprivation, survival conditions, thus diminishing the overall individual fulfillment. Various worldwide associations and different hierarchies of government fraternity are endeavoring to offer the necessary assistance in eliminating the infection impacts but unfortunately standing up to the non-appearance of resources and expertise. In contrast to all other pandemics, Coronavirus has proven to exhibit numerous requirements such that curated appropriation and determination of innovations are required to deal with the vigorous undertakings, which include precaution, detection, and medication. Innovative advancements are essential for the subsequent pandemics where-in the forthcoming difficulties can indeed be approached to such a degree that it facilitates constructive solutions more comprehensively. In this study, futuristic and emerging innovations are analyzed, improving COVID-19 effects for the general public. Large data sets need to be advanced so that extensive models related to deep analysis can be used to combat Coronavirus infection, which can be done by applying Artificial intelligence techniques such as Natural Language Processing (NLP), Machine Learning (ML), and Computer vision to varying processing files. This article aims to furnish variation sets of innovations that can be utilized to eliminate COVID-19 and serve as a resource for the coming generations. At last, elaboration associated with future state-of-the-art technologies and the attainable sectors of AI methodologies has been mentioned concerning the post-COVID-19 world to enable the different ideas for dealing with the pandemic-based difficulties.

## I. Introduction

The evolution of coronavirus disease 2019 (COVID-19) pandemic has drastically altered everyone’s life present across the world. It has spread to more than 200 countries in the third week of August (August 19, 2020) leads to more than 22 million confirmed cases and 780000 deaths worldwide [1]. Compared to approximately 2500000 confirmed cases and 100000 deaths in mid-March Impact on lost lives such as explosive speed and short time is unprecedented [2]. The non-appearance of any viable and general restorative operators and the absence of immunization to create insusceptibility against COVID-19 are making the populace vulnerable to such pandemics. Owing to the pandemic, many fields such as tourism, logistics, etc. are either struggling under financial pressure and the risk on employees to reopen in a new nature.

This global concern is leading researchers and companies globally to develop solutions to ameliorate the effects of the pandemic. Under these huge crisis, cutting edge technologies are emerging to meet the current needs and are becoming essential on a daily basis. Perhaps, it’s far pretty exciting to get up to date when it comes to today’s era cutting edge technologies, various studies are still required to understanding these exquisite practices. We, therefore, aim to provide an elaborate overview of these potential technologies which surely helps to provide a wide variety of knowledge to fight against the coronavirus disease.

### A. Economic Crisis

**FIGURE 1.**
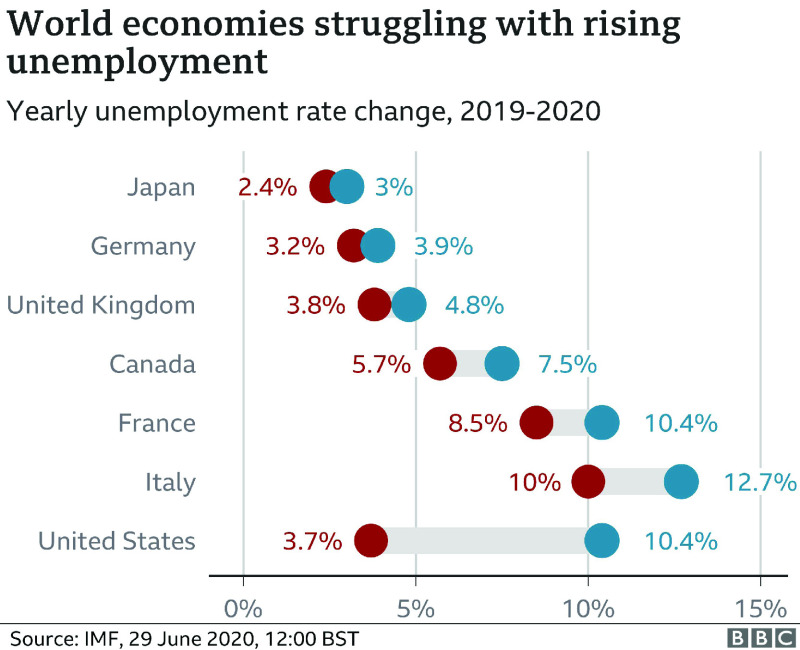
World economies struggling with rising unemployment.

**FIGURE 2.**
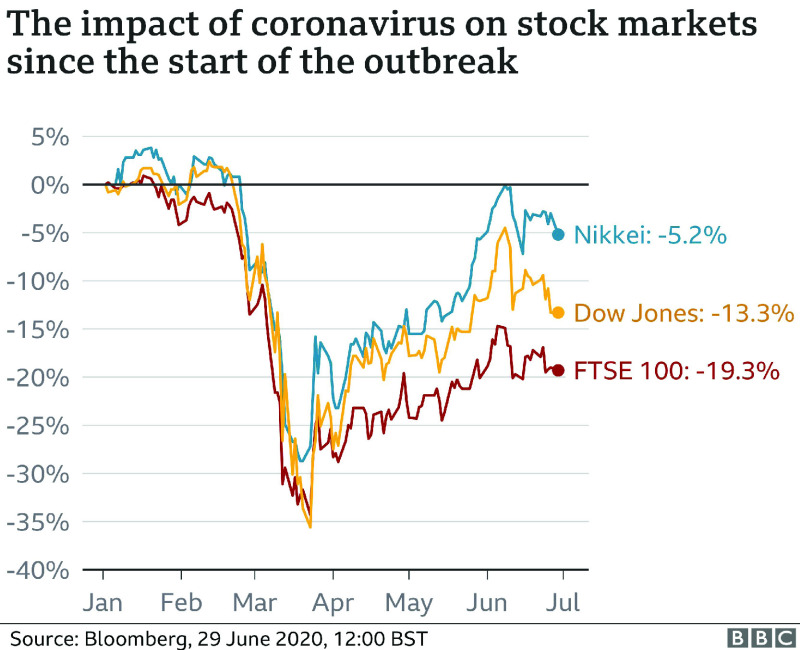
The impact of coronavirus on stock markets since the start of the outbreak.

The COVID-19 emergency is unleashing destruction on work markets in developing economies as shown in Fig. 1 and 2. Enormous scope of people are either getting dropped by their positions or prying significant decreases in their monetary. Several companies with practically zero resources are without a doubt to undergo penniless. The case is as yet advancing and genuine realities don’t get shown up of how this stun has created much decimation.

Gauges show that the joblessness in April 2020 in India took off over 20 percentage. In Brazil, the rate of unemployment will probably increase to an untouched 14 per cent, while in the case of Nigeria, specialists anticipate that the unemployment rate should increase to around 30 percentage. In the interim, about 2.1 per cent of the workforce, have lost their positions or have been compelled to apply for leaves. The legislature has cautioned that work misfortunes could ascend to 5.2 million. In Mexico, 350,000 conventional positions were lost in the month of March, and is quickly expanding. In the Russian Federation, joblessness strongly expanded, and much issues like significant compensation unpaid debts, decreases in wages and moves to low maintenance work were gradually scene. These employment misfortunes are just one element of the unfurling emergency in rising countries.

**FIGURE 3.**
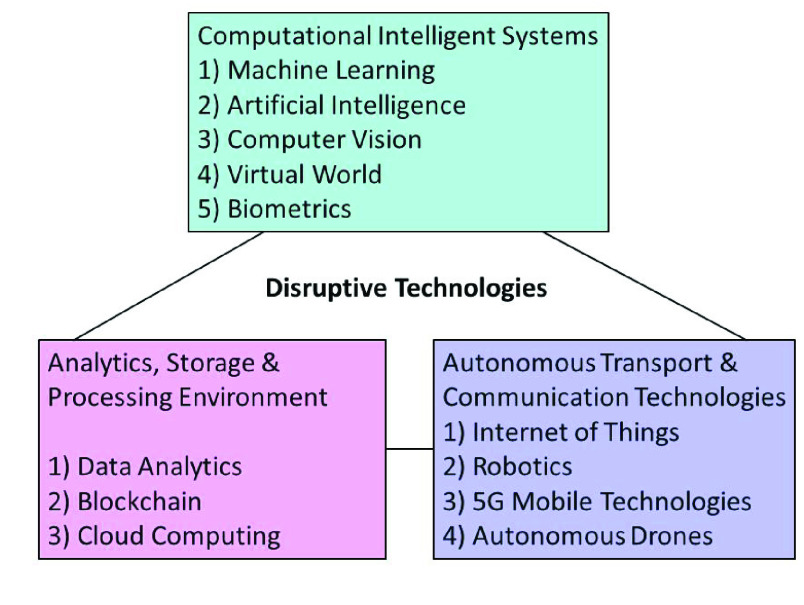
Categories of disruptive technologies.

During the most recent decade, it is obvious that policymakers are keen on the transformation of advancements which eventually leads to an innovation funnel with different innovations. Emerging technologies are radically innovative, growing at a steady base and gaining prominence over time, which-in turn induces a substantial socio-economic impact. As innovation appropriation rate is accelerating step by step, future advancements are consistently improving the current work so that these gradual improvements influence the different networks included otherwise. Cutting edge innovation can prompt the progressive change that evacuates an accomplished innovation and produces new ventures, markets and worth organizations that in the long run can transform a current way of life [3]. The expression “Disruptive technology” is going to get used at various sectors which in turn incorporates a significant change in the mechanical use and design after some time. Similarly, we are assimilating various likewise technologies together, thus, attempting to plan advancements concerning the battle against COVID-19. Fig. 3 gives a few instances of disruptive advancements in the course of recent years. Before starting the use of any inventive innovation, endeavour pioneers should grasp the setting which incorporates calibrated interconnections among individuals, cycles, administrations, and conditions. There are explicit patterns that will drive noteworthy changes and openings throughout, particularly in the zone of foundation and activities [4].

We estimate that people under the age of 20 are half as likely to be infected, and in adults over the age of 20, clinical symptoms appear in 21% of 10 infections (95% possible interval: 12–31%). 69% (57–82%) of infections occur in people over 19 and over 70 years of age [5]. Accordingly, we found that child-targeted interventions may have a relatively low impact on reducing SARS-CoV-2 transmission, especially if the spread of subclinical infections is low. As a result of population differences in different systems, our estimates of vulnerability to clinical fraction and age affect the expected global burden of COVID-19. In countries with a younger demographic structure, such as many low-income countries, the number of individual medical cases may be lower than in older populations, although comorbidities in low-income countries may also affect the severity of the disease. Without effective control measures, relatively old populated areas may experience a high incidence of COVID-19, especially in the later stages of chronic infection.

It is essential to address the effects of the pandemic on the Works in many sectors including aviation, automobiles, pharmaceuticals, banking, consumer electronics and tourism. Table 1 speaks about the various kinds of innovation alongside the rising advances. To cover the expansive scope of developing, future and disruptive advances to battle COVID-19, following are our commitments: First, we present a short outline of the pandemics that have happened in the previous century highlighting recent pandemics and associated technology innovations helping us today. Second, we reviewed the evolving nature of technologies to combat COVID-19 and other pandemics wherein emphasis on the inherently disruptive nature has primarily been considered. Third, we identified a technology-enabled COVID-19 use case using Smart AI Intelligent technique the diagnosis task. Fourth, we summarized the challenges, priorities and future directions in each area. Finally, we concluded our results with some unexplored open research questions which need to be addressed in the near future.

**TABLE 1:** Applications of Emerging Technologies in the COVID-19 Pandemic

S.No	Emerging Technology	Applications in the COVID-19 Pandemic
1	Artificial intelligence (AI)	AI can help in the screening, tracking and predicting the current and future patients [5], [6]. Early detection and diagnosis are primary examples of AI usage in pandemics especially upon the availability of annotated datasets.
2	Cloud computing	Remote work and education are becoming more common to accommodate lockdowns. Cloud computing is essential to support real-time streaming and video conferencing for collaboration.
3	Telemedicine	Through video streaming and virtual reality, patients can interact with medical professional about any health conditions avoiding hospital visits.
4	Blockchain	The algorithms provide real-time information about the pandemic to all strategic partners, diagnosis disease management processes. Blockchain can help in managing the vital supply chains.
5	5G + smart applications	5G technologies along with medical sensors and smart applications enable real-time data processing for remote health monitoring allowing access of information by medical staff.
6	Internet of things (IoT)	IoT devices connected to the Internet in hospitals and strategic locations can provide context awareness. In addition to monitoring patients, IoT services can help in monitoring crowds, supply chain monitoring, and mobility monitoring at the national level.
7	Drones	Drones can help in the surveillance of large or crowded geographical areas and disseminating knowledge and information to the public. Drones can also be used for delivery of product with limited interactions to reduce the spread.
8	Robotics	Robotics can be used for disinfecting shared areas, such as hospitals and work places to reduce the spread. It can be used for interacting with patients and performing simple tasks to protect employees in different sectors.

The rest of this paper is organized as follows: In Section II, we present a brief overview of the pandemics that have happened in the previous century. In Section III, we present the source, theoretical subtleties and exploration status of different developing and future advances. The use cases based on smart intelligent technologies, to diagnose COVID-19 is presented with results in section IV. Section V examines technological advances, basic difficulties and up and coming open doors in this specific context and Section VI concludes the paper.

## II. Pandemics and Technology: a Historical Perspective

### A. Pandemics History

In the last century, there were numerous outbreaks and pandemics, such as Spanish flu, Asian influenza and Hong Kong flu. Although, most of these outbreaks had been caused by corona viruses Various influenza viruses such as SARS-CoV and MERS-CoV, such as H1N1, H2N2 and H3N2, have evolved over the past 105 years. Spanish flu is known as the most dangerous epidemic in human history, with more than 50 million deaths [7]. The disease is caused by an H1N1 infection that is believed to have started with algae. Unlike most diseases, Spanish flu has unique characteristics that are dangerous to young and healthy people due to which the virus causes a cytokine storm in the immune system of the patient, which often leads to death [8]. Further, the Asian influenza pandemic began in Singapore in February 1957. It was the second major pandemic of the 20th century. The death toll in the United States only was estimated around 116,000, and a total of 1.1 million worldwide were infected [9]. The virus has been identified as type A1 H2N2 virus, which is believed to be of bird lineage similar to H1N1. Eleven years after its inception, the H2N2 virus strain no longer infects the human host. The Hong Kong influenza pandemic in 1968 and 1969 is the third dominant flu of the 20th century. It is caused by the H3N2 virus, which is thought to be derived from the H2N2 virus, which also caused the Asian flu pandemic 1957–1958. Most of these pandemics subside on their own and disappear after being active for few years. The main lesson to handle future pandemics from a technology point of view is to prepare for the long run and develop techniques to detect, diagnose, and control the outbreaks until a vaccine is developed or the disease subsides in intensity with weaker strains.

### B. Recent Pandemics-SARS-COV, MERS-COV and Swine Flu

COVID-19 after SARS-CoV in 2002 and Middle Eastern respiratory coronavirus (MERS-CoV) in 2012 marked the third largest corona virus infection in the 21st century [10], [11]. Some studies have found that SARS-CoV-2 is caused by the beta corona virus, and is the host of the genetic predisposition that is supposed to cause the bat. SARS-CoV uses a virus-like receptor that modifies angiotensin rather than catalyst 2 (ACE2) and spreads mainly through the respiratory tract [12]. In addition, the Middle Eastern respiratory disease corona virus (MERS-CoV) is a growing infectious disease associated with acute respiratory disease, first distinguished in 2012 in Saudi Arabia. As of 30 October 2018, the WHO had warned of more than 2254 lab affirmed instances of MERS-CoV contamination from 27 nations, including 800 passings [13]. MERS-CoV is one of the high-need microbes recognized by the WHO R and D Blueprint as a result of its high casualty rate (35%) for critical cases, the substantial topographical scope of the store and absence of clinical countermeasures, with primary information holes in veterinary and human the study of disease transmission, insusceptibility and pathogenesis [14].

There is limited evidence of the spread of the disease in the child population. Statistics MERS-CoV is more common in men than women. The frequency of accidents in men (52%) was found to be higher than in women (23%). Men with a history of serious illness are more likely to be infected. In addition, the duration of the disease in adults is 60 years. No currently approved vaccines or drugs have been developed against MERS-CoV [15]. There is no evidence that the infection has developed a respiratory disease and that pork from H1N1 infected pigs may have incurable disease [16], [17]. Evidence that this new strain may be passed from person to person has prompted the World Health Organization to elevate the epidemic to stage 5, which is a strong indication that an epidemic is imminent, and the use of association, correspondence and agreed moderate measures is short-lived. Therefore, this spread was increased to stage 6, which proves that the global epidemic continues. U.S. Department of Disease Control and Prevention(CDC) estimates that the virus alone has caused 43 million infections treated in 195,086 hospitals and the number of deaths worldwide has exceeded 151,700 [18]. In all these pandemics, developing a medication or vaccine takes years. Given the absence of a particular immunization or proper medication, measures had concentrated on recognizing, rewarding and disengaging individuals who have the infection and instructing the general population of ways to reduces outbreaks. This survey discusses the technologies developed to combat recent pandemics as these technologies and associated procedures were critical in absorbing the COVID-19 initial wave of outbreaks in Taiwan, Korea, Singapore, and to some extent China. We have a some examples of the developed technologies during the recent pandemics in Table 2.

**TABLE 2:** Technologies Used to Combat Past Pandemics

S.No.	Recent Pandemic	Technology used with their use cases
1	SARS-CoV-2	The Shanghai Institute of Technology and Physics have created an infrared sensor that can measure an individual’s temperature without physical contact. The sensor assisted with finding broad and simple application to check the spread of SARS outdoors, for example, in railroad stations, air terminals, and harbours. It was seen as a ready to forestall cross-disease by checking the patient’s temperature [19].
		Emergency clinics in Taiwan utilized the access network processing innovation, which was created by the Futures Laboratory at Argonne National Laboratory (Argonne, IL, USA) to share and safely store X-beam pictures and clinical data about patients. The matrix permitted therapeutic services experts to distantly see records and team up on analyzing and making clinical choices. It associated the medical clinics with one another, and with the WHO and the US Centers for Disease Control [20].
		Singapore’s DSO National Laboratories has built up a program that models the spread of SARS utilizing software models of PC security programs that track PC infections across systems. The framework included different information about the population, such as, age, well-being status, and recurrence of contact with others into an electronic stream diagram that indicated how every individual connects with others. The factors were even adjusted to exhibit how changes may influence the spread of SARS [20].
2	MERS-CoV	During the MERS emergency, Singapore General Hospital presented an online physiotherapy program permitting physical advisors to distantly screen patients in their homes. While utilizing the webcam, patients were made to speak with their advisors, who can, thus, show their patients new activities and give them input on their advancement [21].
		Sunday Communications, a Hong Kong cell phone provider, provider clients with a notification service if they are close to contaminated areas. Clients that subscribed had their telephones tracked and would be notified by SMS at locations (within 1 km) where SARS cases were reported. It conclusively demonstrated that this framework forestalled MERS cases later [21].
		In Munster, the medical clinic utilized its ongoing finding framework identifications close by Amazon EMR’s that encouraged all emergency clinic staff, patients and guests to determine who had come into contact with the tainted patient. This innovation was enhanced by the emergency clinic’s video observation framework to guarantee no contact with the patient was neglected, as indicated by the report [22].
3	Swine Flu Influenza	A Korean based organization utilized a FluChip innovation to keep patients out of the emergency clinic and hold space for individuals who are genuinely in worse conditions utilizing chatbots on the medical clinics’ sites to help concerned patients decide if their side effects are likely associated with the swine flu infection and their hazard level. It associated high-hazard people with the side effects reported to ensure the correct degree of care. If the framework recognized somebody in the ER with an affirmed flu infection case or expected case dependent on pandemic hazard, those patients are provided technology for remote monitoring including pulsometers and thermometers to follow their side effects from home. The data assembled was then revealed through the telehealth platforms, and nurses worked nonstop to ensure the manifestations do not advance [23], [24]
		Another methodology was applied by the Swedish organization MedDay, which recommended that individuals enter side effects into PDAs or cell phones, which would remotely transmit their data and subsequently, could aggregate and screen this information. The organization asserted that this could be utilized as an initial notice framework across the nation for episodes of maladies, synthetic assault, or different ailments [25].

## III. Impact of Emerging Technologies and Future Technologies on COVID-19 Pandemic

The following sections discuss in details emerging and future technologies to combat COVID-19 and other pandemics in general.

### A. Artificial Intelligence

**FIGURE 4.**
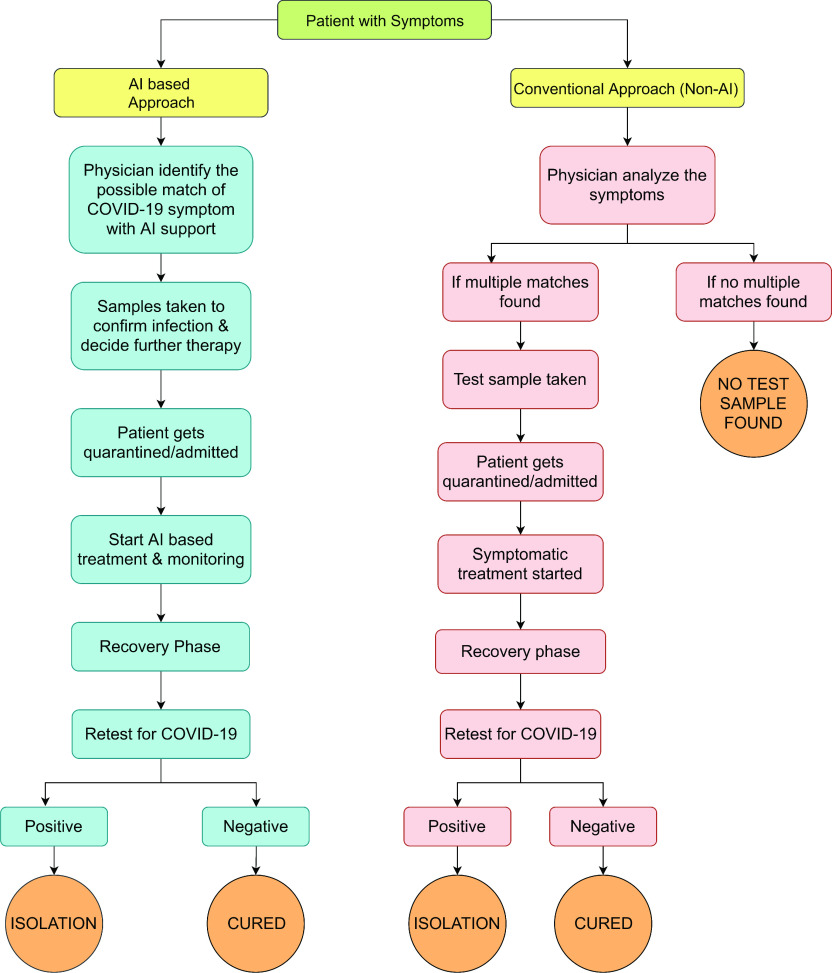
General procedure of AI and non-AI based applications that help general physicians to identify the COVID-19 symptoms.

Artificial intelligence is a method that can analyze the spread of the infection, distinguish high-risk based patients, and be compelling in controlling the illness continuously. It can foresee passing dangers by sufficiently investigating information from past patients. Moreover, global patient help with suggestions for populace testing, clinical consideration, notice, and disease control [26]–[28] can assist with battling this pandemic infection. This innovation can possibly improve the arranging, treatment and results of COVID-19 patients, making it a clinical proof-based apparatus. Fig. 4 illustrates general training for applications associated with AI to help general practitioners identify the symptoms of COVID-19. The chart shows and compares the lowest non-AI treatment flow with AI-based therapy. It also demonstrates the vital role that AI plays in to implement the high resolution-based applications, which undoubtedly reduces complexity and time. Moreover, it guides various doctors to not only focus on treating the patient, but also on controlling the disease through several artificial intelligence-based techniques. The process of AI follows up with key scores and tests which are analyzed with high accuracy to reduce the total number of actions taken during the entire process and thus, makes it more reliable.

#### 1) Outbreak Size Estimation

AI COVID-19 can fight the proliferation of coronavirus outbreaks by analyzing people’s phone usage patterns. The practice can also be extended towards people who become sick, dead or take care of their family due to a coronavirus infection [29]. For example, if someone accesses Wuhan’s wireless carrier data from November 2018 to March 2020, then one will find vast phone’s operating patterns. The phone trend includes abnormal call usage by sick persons in the early mornings, non-telephone activity of people dying of coronavirus infection, or new phone calls from people present in closed cities. In this scenario, AI can analyze various functions [30]. For example, the ML model can predict users with different customized functions through phone application logs [31]. Deep learning (DL) will be a promising AI-based technology with various high-performance prediction models to accurately forecast mobile app usage, such as abnormal call behaviour and phone service inactivity [32]. which plainly reveals users’ patterns, and allows the company to estimate the extent of coronavirus outbreaks. Mobile operators can track the movements of patients and predict their occurrence from 500 cities in the United States while considering user privacy [33]. AI can analyze user operating systems to evaluate how crowds are still gathering and to indicate specific geographically isolated areas. Meanwhile, some users in China use mobile phones for public transport identification every day so Chinese use AI to track customer movements and COVID-19-infected passengers [29], [34]

#### 2) Coronavirus Detection

AI can come up with a solution considering the characteristics of facial recognition application where COVID-19 can be detected by checking the temperature of a person’s face. The blockchain-AI architecture for coronavirus combating COVID-19 architecture is shown in Fig 3. The study in [35] indicates an deep learning, the face recognition models get trained to accurately determine if a person is wearing a mask. Unlike these studies, the authors of [36] rely on respiratory in coronavirus detection, it is critical to accurately identify people’s unexpected abnormal breathing patterns in a distant and uninterrupted way [37], [38].

Chest CT image analysis is another method to detect coronavirus-positive patients [39]. Non-restrictive chest CT scans have shown to be an effective tool for diagnosing, measuring and monitoring diseases such as coronavirus. With a large number of chest CT scans, a diagnostic system was proposed which combines powerful chest CT function with greater accuracy. Further, CDS Imaging analysis was performed on COVID-19 patients on an initial screen [40]. The results indicated that the computed tomography (CT) image of COVID-19 has some distinct characteristics as compared to different types of pneumonia. The study of [41] also used image analysis-based techniques for coronavirus detection. As confirmed in the simulation, the ResNet 50 model was able to provide better Performance. Tests using the 2019 nCoVR dataset showed SARS-CoV-2 and correct classification performance while distinguished from other coronavirus strains such as MERS-CoV and SARS-CoV. On the other hand, the research effort of [42] directed to verify the diagnostic uncertainty of COVID-19 detection. Later, the Bayesian convolutional neural network (PCN) was considered to estimate the uncertainty of the TL solution. The reason for this examination was to improve the symptomatic presentation of human-machine combinations utilizing the openly accessible COVID-19 chest x-beam dataset. Another interesting task of [43] was to present a system for detecting coronavirus COVID-19, using an internal smartphone sensor. The proposed model allowed people to get identity with viral pneumonia, which can possibly discover coronavirus-related symptoms by reading the signal measurements from the smartphone sensor and then scanning the CT image. It can also be useful for radiologists with smartphones to track the progress of the disease.

#### 3) Coronavirus Diagnosis

Developing innovative and efficient diagnostic, along with therapeutic strategies to combat coronaviruses, has enormous implications [44], [45]. The work in [46] presents the AI-based model, can break down the standardized force of the x-beam picture to describe and depict the patient’s status as a positive or negative COVID-19. Based on the current situation where there is no general COVID-19 data set, the learning-based scheme gets validated with 25 confirmed COVID-19 cases present in 50 chest x-ray images which in turn showed superior performance and high accuracy in terms of COVID-19 detection. DL was used to quantify COVID-19 infection in CT images [47]. The study of [48] introduces an ML model for predicting potential against the SARS-COV-2 virus. Antibody responses were predicted by collecting trained antibodies. References [49] consider an alignment-free basis approach for ultrafast, quantifiable and accurate classification of the entire COVID-19 gene. The results trees combined with supervised learning were used for genome analysis in large data sets. Besides, a study by [50] identified miRNA-like hairpins that could infect the COVID-19 virus.

#### 4) Vaccine/Drug Development

In some rapid studies, AI techniques can be an attractive tool to support vaccine production. The HLA-binding prediction tool using peptide stability analysis gets mentioned in [51], therein 777 peptides were predicted as suitable binders in 11 MHC isoforms with high predicted binding scores. Preliminary results can play an essential role in designing effective vaccines for COVID-19. A study carried out in [52] insight-based generation network complex model gets obtained to generate potential 97-NCOV drug families derived from HIV-1 proteolytic enzymes [53]. They suggested that the antiretroviral drug atazanavir, which has been used against HIV, might be valuable for creating specific compounds for COVID-19. The efficacy of the detected drug is in the testing phase before patients with coronary artery disease can get a chance to use it. Further, the study of [54] combined ML with a statistical analysis approach to design a data-driven drug relocation framework by systematically mining large-scale knowledge maps and organizational data.

#### 5) Prediction the Future of COVID-19 Outbreak

The recent emergence of the COVID-19 has highlighted the need to predict various unfavourable future events. COVID-19 pandemic models were considered for predicting and controlling outbreaks of prevalent coronavirus [55]–[57]. Along with it, AI has recently been used to predict the spread of coronaviruses. For instance, a predictive model was equipped that uses AI to estimate the size, length, and endpoint of COVID-19 across China [28]. Automatic encoders were designed to model pandemic transmission dynamics based on data sets collected from WHO sources. Clustering methods were greatly used to classify multiple cities and regions to investigate the spread of the fatal virus. As reported in [58], another model for predicting explosions was developed in Mexico. Probability models based on the biological characteristics of the virus, the pathophysiological mechanisms of the disease and the Markov chain were primarily considered to indicate the probability distribution of disease cases in the state of Mexico. It also estimated the number of actual cases in Mexico which certainly helped in assessing the outbreak of viruses. At last, this study provided us with some appropriate actions for any future outbreaks that happen in Mexico. During the statistics gathered from the COVID-19 outbreak, a study in China used ML to develop predictive models for future infections [59]. In the meantime [60], the authors obtained better probabilistic insights into the data distribution about the outbreak and DL networks did not determine fuzzy rule derivatives. Based on the results of the model, policymakers can now see a better picture of the outbreak of coronavirus and assess similar possibilities in the future. On the other hand, the virus was tested in DL [61] to develop a host prediction model to identify how the virus-like mink virus spreads to other sources and determine its relationship with COVID-19. Thus, this study traced the origin and spread of the coronavirus and nonetheless, provided appropriate preventive measures. Therefore, the prime focus is that an AI-enabled service is to spot corona patients accurately as shown in the Fig. 4.

#### 6) Challenges

AI can assume a major function in decreasing the effect of irresistible COVID-19. However, current AI systems are still in their early development, and the following are the several challenges and barriers that prevent the use of artificial intelligence in managing the effects of COVID-19.

• While accomplishing solid and exact outcomes, Artificial Prototypes are needed to have enormous preparing information. Perhaps, because of the uncommon idea of the pandemic, it has been an insufficiency of data onto which AI models are make ready, which in turn has delivered a couple of AI models inefficient [61].
• Not simply the absence of open information prevents the exhibition of AI Prototypes, but an excessive amount of boisterous and exceptional information also poses a significant challenge towards the use of artificial intelligence techniques.
• AI, in its present structure, has some different restrictions that depend on human information. Human aptitude is expected to manage the execution of computerized reasoning advancements which can, in the end, have a major effect in the battle against COVID-19 disease. In spite of a couple of challenges standing up to the AI Prototypes, their promise to battle against the COVID-19 pandemic cannot be dismissed. Recently, AI development emerged with fabulous innovations in the areas of ML, NLP, data inspecting, etc. This enhancements promise to show the ability of this technology in combating the COVID-19 pandemic organization structure.

### B. Cloud Computing

In the last few months, the COVID-19 pandemic has damaged the lives and businesses for an unprecedented number of people. Nevertheless, if there is a substantial part which revolves around this phenomenon, then it would be the cloud computing sector. From a Facebook post to Netflix, this quarter reports twice as many new subscriptions as expected this year, which in reality, gets experienced only on New Year’s Eve. Also, the Internet and encompassing environments have gotten more important than any other time. To investigate more about this, we will take a gander at how the quickly developing COVID-19 pandemic has influenced the cloud business and how suppliers are reacting to the developing interest in the basic infrastructure foundation.

#### 1) Productivity Away From the Desk

As physical communication is no longer an acceptable form of communication in the light of social distance initiatives, companies around the world, have now dramatically transformed digital solutions to maintain productivity scope. Furthermore, for many developed countries, including Europe and the United States, employees working in key business areas must work for an unlimited number of hours to normalize the company interest. Schools and universities have started to develop video conferencing platforms to facilitate distance learning through direct or registered lecturers.

As a result of all these factors, it is not surprising that joint solutions such as Slack and Zoom announce the highest number of growth indicators getting recorded over the past few months. According to a Microsoft blog post for March, Italy’s monthly usage increased by 775% after orders because its collaborative product across the country remained intact with other service products. The company’s virtual desktop offer also had a small 300% service pump [62].

#### 2) Delivering to the Needs of Millions

Meanwhile, the entertainment industry has greatly enhanced self-isolation or isolation from people. After the cinemas got shut, many changed to choices, for example, Netflix and Amazon Prime Video, the first observed application download expanding by the at least rate of rate 60% in Italy, 30% in Spain and 9% in the United States [62]. Numerous media, for example, computer games are likewise picking up notoriety. According to Verizon, a US-based telecommunications company, video game usage has increased by about 75% during peak week after isolation. According to Nielsen’s Super Data Research, consumer spending on digital gaming increased by 11% compared to March 2019 [62]. At long last, toward the beginning of April, the Amazon Twitch computer game streaming site arrived at another record of 1.7 million watchers in a single game. These stuns to online amusement opportunities to make it impossible for many Internet service providers around the world to increase traffic. To this end, regulators in the EU and India have appealed to well-known streaming websites, including Netflix, Amazon and YouTube, to lessen the quality nature of their streams for high-definition content temporarily.

#### 3) The E-Commerce Boom

E-commerce is an additional industry where many people far and wide go to webpages like Amazon to purchase staple goods, regular fundamentals and clinical requirements. As per a study by Nielsen, patterns demonstrate that soon, customers will shop online as they will attempt to bar themselves willfully.

#### 4) Growth Opportunities for Cloud Service Providers

It is important to note that giants like Amazon, Google and Microsoft determine their incomes and development from their individual cloud organizations as well as from partner administrations, for example, internet business and programming licenses. Thus, it is impossible that COVID-19 will significantly affect the net benefit of these organizations. In addition to the urgent need for remote work created this year by COVID-19, it is much obvious that organizations from different enterprises are currently starting to understand the advantages and estimation of cloud-based computing. Therefore, numerous organizations will begin to scale their advanced endeavours and put vigorously in data innovation and cloud assets in the coming years. Capturing at least one part of this unused market will allow smaller providers to enter the cloud industry, which is currently dominated by Alibaba and Google. However, finding and accessing a business on the brink of digital transformation is a severe obstacle for many cloud service providers [62] These organizations have been safeguarded by admittance to crystal-clear and efficient data in the worldwide startup system. Further with its crucial streaming stage, OddApp offers patented solutions that include Oddapp Score and Benchmark Rating (PV) metrics, which address early health and barometer scores. While the outcome of the pandemic is unclear, companies and individuals are discovering the isolated and socially distant practices needed for technology, and its recent advances have prevented the world from realizing its full potential. The cloud may have been a modest cost for many companies ten or two years ago, but its need today is undeniable.

### C. Telemedicine in Healthcare During COVID-19 Pandemic

In today’s world, COVID-19 is a public health emergency of international concern. Primary care is considered a serious issue during these infections. Lack of locks, safety equipment and hospital facilities may make primary health care services vulnerable to infections among non-COVID-19 patients and health care providers. People with severe and chronic medical conditions such as diabetes, pregnancy, obesity, chronic respiratory disease, heart disease, cancer, and mental illness have major problems. This section analyzes the challenges of providing early health care in developing countries during COVID-19 epidemics and discusses the role of telemedicine in meeting these challenges. Telemedicine can play a key role in reducing the spread of the virus, making better use of time by health care providers, and alleviating mental health problems. Fig. 7 shows an example of such an integrated health system.

**FIGURE 5.**
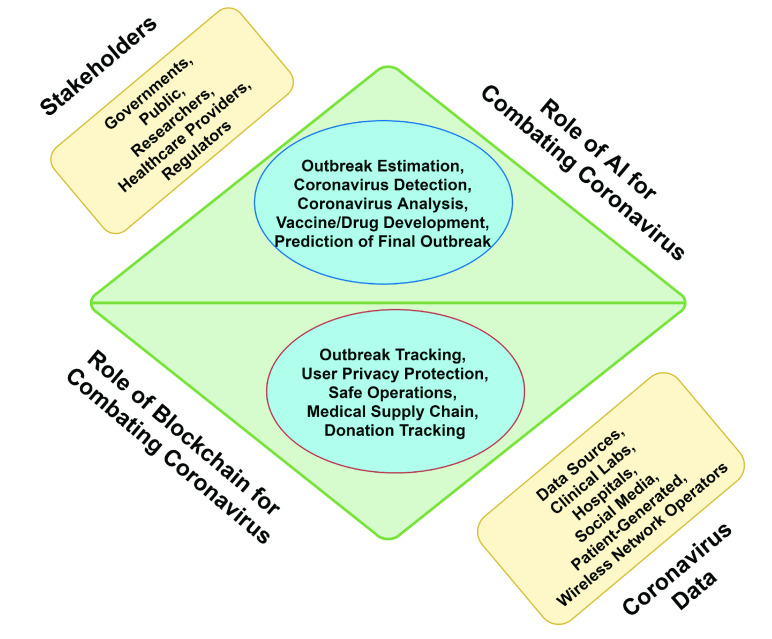
Blockchain and AI for combating coronavirus.

**FIGURE 6.**
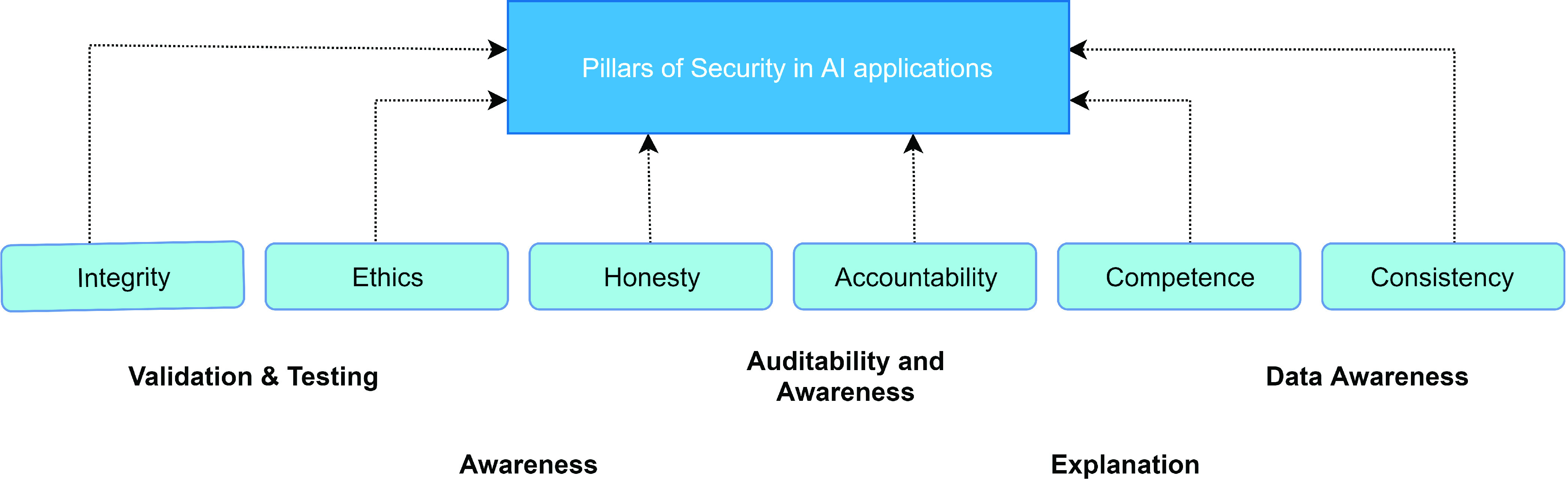
AI security systems.

**FIGURE 7.**
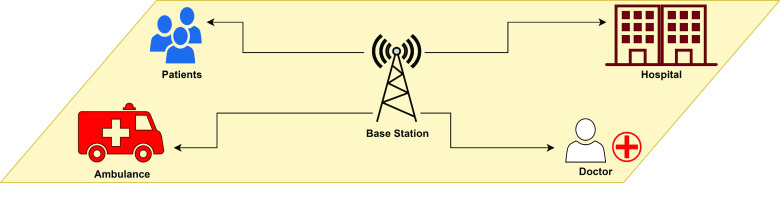
Wireless health monitoring system.

#### 1) Lockdown Limiting Physical Visit to Doctors

Impediments of human development can altogether decrease the predominance of new COVID-19 contaminations [63], [64]. The World Health Organization required “all nations to seek after compelling endeavours to control the infection and hinder the spread of the infection.” Many Countries have impeded and restricted the development of individuals to slow the spread of the infection. Around 4 billion individuals, or a large portion of the total populace, are asked by governments to remain at home to forestall the spread of the lethal COVID-19 infection. Subsequently, individuals with other intense and persistent diseases can’t go to specialists or clinics. Absence of wellbeing tips can expand the wellbeing danger of various social networks. Telemedicine advising can profoundly help these individuals in keeping up a sound life.

#### 2) Risk of Infection for Non-COVID Patients

During the time of the outbreak of COVID-19, visits to hospitals and clinics have increased the risk of infection in patients with non-coronavirus diseases. Testing for COVID-19 in developing countries is very limited by the lack of equipment, which prevents many patients with positive coronary examination from being tested. Many of them are asymptomatic, pre-symptomatic, or mild [65]. Some doctors may even be COVID-19 positive, but not yet tested since they were under the asymptomatic category. Doctors have been reported to be another primary source of infection. Perhaps, those who come to hospitals or medical facilities for health problems associated with coronavirus have a higher risk of spreading infection. As a result, treating non-coronavirus diseases with the help of telemedicine systems can significantly reduce the risk of infection for both the patient and the clinician. In addition, most patients with positive COVID-19 do not need to be hospitalized because the symptoms are mild. Telemedicine health advice is very effective for these patients, as it can curb the spread of infection on a large scale. Most importantly, telemedicine may reduce PPE use, making it more accessible to healthcare providers working with patients having severe coronavirus infection in hospitals.

#### 3) Effective Use of Doctor’s Time

Many healthcare providers are suffering from COVID-19, and they are at higher risk because they tend to perform their duties in hospitals and clinics. Affected professionals must be in a secluded or isolated home. Therefore, in the current crisis, the precious time of these physicians is ineffective if patients are in much need. These home quarantine physicians can make good use of their time by providing medical advice to patients through telemedicine.

#### 4) Relieving Mental Stress

The nervousness about COVID-19 and its impact is extremely upsetting. Numerous individuals are in danger for psychological wellness issues, including wretchedness. Social separation makes it considerably harder. Home telemedicine-based psychiatric counselling can be very effective and useful in combating mental disorders during these infections [66].

#### 5) Strength

• The expanding events of life sicknesses and quick advancement of the applications for media transmission just as IT innovation are assumed as the central point driving business sector improvement.
• Online friend conversations bunches give supportive information. In any case, it is critical to meet individuals experiencing similar strategies that can help with feeling less alone. It is empowering and at the same time, provides much harmony.
• Specialists and various clinical experts can tune in to the talks and get exhibitions of the most recent innovation without leaving the workplace. The kind of telemedicine innovation is essential for medical care authorities who have been working as volunteers in different inaccessible spots.

#### 6) Weakness

• Absence of cognizance of the patients and their affirmation face a couple of issues and troubles in the clinical consideration organizations through telemedicine associations and applications.
• Nonattendance of master types and cutoff making programs.
• One of the significant restrictions is accessibility and expense. The administration may not be gotten to the telemedicine administrations. Telemedicine can open up a few treatment entryways. But, it isn’t viewed as same as block and some specialist office. With the fast ascent cost of medical care, the non-existent in comparable spots, where the necessity is fundamental.

### D. Blockchain-Based Solutions for COVID-19

We analyze the role of the blockchain in the fight against coronavirus using five key solutions which include outbreak monitoring, protecting user privacy, daily safe operations, the supply chain of medical equipment and donor monitoring.

#### 1) Outbreak Monitoring

**FIGURE 8.**
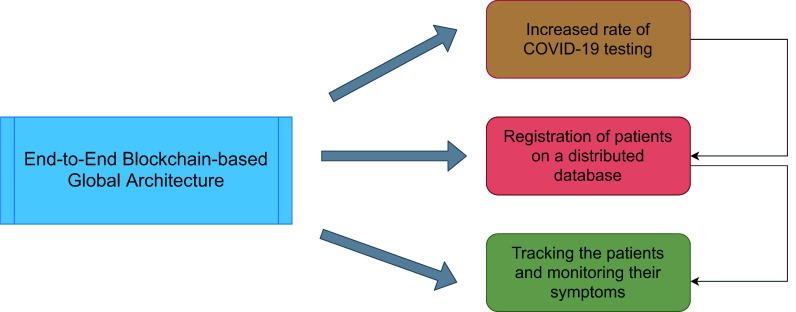
Blockchain-based architecture for COVID-19.

Blockchain may offer potential solutions for monitoring the outbreak of coronavirus. As a matter of fact, blockchain-based data visualization tools can be implemented to track deadly coronaviruses. With the spread of coronavirus disease, blockchain can help thousands of coronavirus sufferers by changing their symptoms of infection [67]. Governments and healthcare organizations can monitor potential patients using blockchain with greater reliability and accuracy at each stage [68]. Besides, blockchain is also useful for monitoring human movement in non-viral regions [69]. The Applications based on Blockchain can be utilized to screen and deal with the COVID-19 patients carefully, consequently alleviating some weight on the medical clinic staff and other medical services work force as shown in Fig. 8.

#### 2) User Privacy Protection

The strategies can help monitor the transport chain by tracking the movements of identified coronavirus victims. However, it may also disclose sensitive personal information to the public, which can cause severe data leakage issues and prevent citizens from working with medical institutions. In an emergency, such as a coronavirus pandemic, the user’s personal information should be compared with other health-related concepts. The value of balancing and maintaining the public interest in fighting the massive virus pandemic with the protection of privacy and human rights is paramount while creating health care policy [70], [71].

The use of blockchain in the coronavirus pandemic allows a community to record patient information like high symptoms, location, historical health status and privacy. Also, data sets are dispersed through a distributed network of governments, health professionals and users [72]. This facilitates all communities to control and monitor any given information such that all data retrieval and data updates are automatically reflected in the network for data flow monitoring.

Blockchains may also provide privacy for coronavirus surveillance networks [73]. Moreover, there are some Asian countries where community surveillance systems have been used to monitor the spread of various viruses. In the same context, using a decentralized blockchain that collects data can not only ensure user privacy but can also be enabled by user nodes to keep private information confidential. It provides the right transparency, accountability and reliability of any given data.

#### 3) Safe Day-to-Day Operations

In the Coronavirus crisis, blockchain has emerged as an excellent platform to reduce the risk of virus infection by performing daily operations in the virtual environment. Here, we consider two popular day-to-day tasks which are user-to-user and cross-border operations.

We can also continue our activities using a digital blockchain-based virtual platform. Current electronic payments between customers and companies can be made via blockchain instead of money, which could be a source of coronavirus spreading [74]. In the Coronavirus Crisis, electronic payments via blockchain may be the best choice for people as they still encourage various businesses. The decentralized blockchain concept ensures payment with a secure, transparent and unchanged interface, and no human interaction. When customers take isolated measures to prevent the COVID-19 outbreak, they can perform contactless bids in remote locations.

#### 4) Cross-Border Operations

The lack of trust, lack of validation, lack of information sharing, and other difficulties in joint ventures while overseeing the national gap [75]. Furthermore, thanks to the decentralized ability of blockchain to hold corporations and public companies with a sustainable exchange such that health support process is always guaranteed. Another challenge the government faces in the event of a massive coronavirus disaster is to stop cross-border delivery.

#### 5) Medical Supply Chain

Blockchain can be beneficial for supply chain applications such as commodity supply chain, commercial supply chain, etc. [76]–[79]. A continuing supply of medicines and foods in this pandemic crisis is a challenge for the healthcare industry. Blockchain technology can help supply chain companies achieve faster supply flow by tracking the source-to-destination flow in a reliable way. Recently, Alibay, along with the Zhejiang Provincial Health Authority and the Department of Economics and Information Technology, launched a blockchain-based platform that allows users to monitor the supply and demand of medical products [80]. It includes the recording and monitoring of coronavirus infections such as masks, gloves and other protective equipment. In the event of proliferation, rapid response and rapid supply chains are the most important weapons for authorities to solve any supply chain issue.

• *Product Requirement:* Provides a solution for real-time demand for fast needs, i.e. supply chain scaling and for refurbishing medical factories.
• *Supply Credibility:* Provides a solution to control product quality on the factory side, such as product specifications and supply.
• *Customs Certificate:* A digitally signed certificate showing negotiations between suppliers and users, which reflect customer behaviour such as purchase and payment transactions with that supplier in the blockchain.

#### 6) Donation Tracking

The potential of blockchains in donor tracking applications has been explored in recent studies [81] and [82]. In the Coronavirus Crisis, donations are one of the most critical measures to support life and medical care for victims. An important issue is how to monitor donor activity to ensure that donated goods are transferred to the target victim. Blockchain may be a viable solution [83]. The donation process can be monitored by a blockchain that provides signatures and certificates for each donation renewal. For example, donated equipment such as N95 masks can be listed down with the related donation level and target recipients [84], [85]. Besides, blockchain allows suppliers, health professionals and NGOs to track the progress of their donations [86].

#### 7) Challenges

A few and non-specific challenges forestall the utilization of blockchain in COVID-19 situations. Before blockchain-based plans can be executed in the current condition, the following issues must adequately get resolved.

• The first non-specialized test to blockchain usage is the absence of mindfulness about blockchain and its latent capacity. Besides, a few people have reservations in regards to the utilization of blockchain since they partner blockchain just with digital forms of money and false exercises.
• In spite of the fact that non-specialized difficulties can be dealt with by expanded mindfulness, the fundamental problems of blockchain execution are the specialized ones. Blockchain-based stages frequently experience the ill effects of their absence of versatility. The current emergency requires the utilization of an exceptionally versatile arrangement since it is practically influencing all individuals around the globe. Right now, just a couple of blockchain-based stages are accessible, and almost every one of them has characteristic adaptability requirements.
• The reaction to the current pandemic requires the union of different rising and heritage innovations. Since blockchain innovation is generally new and juvenile, it gets hard to incorporate blockchain applications with heritage frameworks.
• One of the favourable critical elements of a blockchain and the non-appearance of any focal power can once in a while induce severe blowback. In the upcoming occasions, because of the few advantages that it offers, blockchain innovation can possibly turn into an imperative piece of the human services industry and the fast reaction structure.

### E. 5G Network Technologies

Contrasted with 4G, 5G is required to perform better as far as quicker speeds, lower latency, more broad reach, expanded accessibility and more noteworthy security are considered. 5G network innovation can possibly reform the clinical business with other supporting advances, for example, IoT and AI. The commercialization of 5G innovation in China has just changed the COVID-19 pandemic by offering better help to forefront staff and encouraging progressed infection and patient observation, alongside information assortment and examination of them [87]. Considering China, for instance, COVID-19 talks about the various ways a nation can embrace 5G to improve the adequacy of their endeavours to battle the emergency.

#### 1) 5G+ Telemedicine

The problems can be solved on 5G mobile networks with features such as high-speed delay and high-speed data transmission [88].

• China already has 5G technology which got commercially released in early November of last year. Some of the features that 5G network offers for telemedicine are present in many hospitals, and medical centres in China, where the 5GC telemedicine platform for COVID-19 patients was introduced.
• West China Medical Center launched COVID-19 5GC Remote Advisory Platform with Chinese Assistance telecommunication.
• A hospital affiliated with Kun Kunming Medical University has launched for the diagnosis and treatment of COVID-19 disease [89], [90].

#### 2) 5G+ Medical Imaging

Recently, medical imaging technologies such as PACS (Picture Archive and Communication Systems) have become an integral part of diagnosis and treatment. Emerging cellular networks and next-generation technologies such as artificial intelligence and large data analysis enable PACS to perform advanced data analysis and management with minimal effort. The Wuhan 5G-enabled medical imaging site, a professional field hospital at Liechtenstein Hospital, has reduced the burden on hospital staff to detect COVID-19 patients in real time. [90].

##### a: Cloudminds’ 5G Robots in Wuhan

Recently, a field hospital with 5G-enabled multi-smart robots was opened in Wuhan, China. Hosted by a company called Cloud Minds in Beijing, the robot can clean, disinfect buildings, deliver medicines to patients, and measure temperatures. The facility, commonly known as the Smart Field Hospital, used a variety of IoT devices to reduce the burden on hospital staff and to monitor their vital signs, such as temperature, heart rate and blood oxygen levels, without having them to attend to healthcare workers physically [91].

##### b: Patrol Robots in Multiple Cities of China

A robot organization settled in Guangzhou, China as of late planned the 5G police watch robot through the Edge Computer MIC-770 created by Advantech. Conceived at the convergence of AI, IoT, 5G and cloud computing innovation, the shrewd robot is outfitted with five infrared thermometers and a high-goal camera to gauge the internal heat level of 10 individuals all at once. Likewise, utilizing an ecological sensor, police can check whether anybody gets covered by this robot. At the point when a robot experiences an individual who doesn’t wear a mask or has high internal heat level, it quickly cautions nearby specialists [92]. The robot has been utilized in numerous Chinese urban communities, including Shanghai, Guangzhou and Guangxi.

### F. Internet of Things

Today’s sophisticated IoT products may play an essential role in controlling the spread of viruses and treating infected persons. By implementing touch-free visits, various health compliances and body temperature monitoring in one place, these products support the collection of valuable data when used in offices, warehouses, hospitals and more.

Further, products such as touch-free tablet delivery and touch-free heart rate measurement are positive features for hospitals.

#### 1) Tracking the Coronavirus Pandemic

A study conducted by the Massachusetts Institute of Technology (MIT) suggested that by overlaying geographic information systems (GIS) on IoT mobile data, epidemiologists may be able to identify those who are infected. Thus, this technology can help monitor high-risk patients and provide useful data to medical personnel.

#### 2) Connected Thermometers

While considering hospitals, connected thermometers are getting used to monitor body temperature and report real-time differences. With the help of the IoT Access Controller, these devices transmit real-time patient data from sensors to nurses for continuous monitoring.

The data collected from more than one million thermometers are outlined every day to a map of the United States, indicating that the heat is increasing. A team of product consultants in India has designed an appropriate injury detection system that remotely measures the skin temperature of a person passing through the camera using AI-based thermal imaging.

#### 3) Smart Wearable

At Istituto Italiano di Tecnologia, researchers designed a sensor suite to monitor human body parameters which alert the user whenever the body temperature rises above 37.5 degrees. In China, synchronized bracelets and rings are used with the AI platform, which is capable of constantly monitoring vital signs, including temperature, heart rate and blood oxygen levels [93].

#### 4) IOT Buttons

The first Internet of Things button was used in a hospital in Canada. It was recently designed which primarily addressed the need to quickly deploy upon all sizes of facilities to instantly alert managers of cleaning or maintenance problems that could endanger public safety.

### G. Global Use Cases of Drones to Fight COVID-19 Pandemic

Different robots address exceptional direct/backhanded areas, decrease critical holes in assets, improve versatility, and extend its usefulness to check COVID-19. Inferable from the significance of robots and their capacity to help the nation’s endeavors to battle the COVID-19 pandemic, individuals have begun to use them in numerous nations around the globe. Various sorts of supplement portray the different use instances of government-based robots in the battle against the COVID-19 pandemic. Fundamentally, there are two kinds of utilization cases concerning drones which are:

• Use cases of drones to fight COVID-19 infection.
• Drone use cases (support action) during COVID-19 infections.

#### 1) Drone Use Cases in Combating COVID-19

##### a: Spraying Disinfectants

Robots which were intended to shower pesticides on farmland have been re-architected to clean huge regions [94]. Nonetheless, the viability of utilizing drones for cleansing has not been resolved [95], yet drones are a basic utility which is generally utilized in nations battling the COVID-19 pandemic. Robot organizations in nations like China made different coordinated efforts with agrarian exploration foundations to utilize drones successfully for sterilization. South Korea, the United Arab Emirates, Israel and India have likewise begun to draw in robots to shower pesticides in metropolitan and rustic zones. The essential recipients of robot based purification tasks are government workplaces, emergency clinics and public spots [96].

##### b: Monitoring Body Temperature

Government officials around the world use drones equipped with thermal cameras to measure body temperature in public places. Some drones are equipped with artificial intelligence-enabled cameras that detect abnormal body temperature [97]. According to a study by the University of Southwest Australia, drones furnished with special cameras have improved their ability to detect coughs, sneezes, human heart, respiratory rates and thus, helps in monitoring the complete body temperature [98]. Countries such as China, Saudi Arabia, Jordan, Israel and Bulgaria are using drones for large-scale body temperature monitoring [99].

##### c: Medical and Food Supplies Delivery

Social distance is considered an essential means of preventing the spread of COVID-19. The existence of public alliances in public places has dramatically reduced the efforts of governments around the world, but there are some essential human activities that people must undertake by themselves. For example, depending on their daily needs, people go out and buy food and medicine patients with COVID-19. During this test, the drones were individually designed to meet specific needs such as ship board food and medical supplies. The benefits of using drones to transport food and medical supplies from COVID-19 are particularly because air traffic congestion is significantly reduced. Drones can carry medicines, medical equipment and even blood samples. Antwork has transferred medical samples and individual items from New County People’s Hospital to the new Military Disease Management Center [100]. The company has considered expanding its business in Hangzhou and Wuhan, China [101]. Chinese e-commerce company named JD used drones to deliver medical equipment to hospitals located outside 34 Km [102]. China has been reported that robots are getting widely used in hospitals to meet the needs of nasal congestion patients. Additionally, the robots have also started to feed patients in COVID-19 infected wards. Drone company Zipline has already demonstrated and tested drone-based medical products in Rwanda, Africa [103]. The company is currently seeking permission to start a business in the United States [104] in consultation with the U.S. government. Similarly, the Canadian company named Drone Delivery Canada has begun discussions with the Canadian government on drug distribution in rural areas using calibrated drones [105].

#### 2) Drones Use Cases During COVID-19 (Supportive Activities)

##### a: Surveillance and Ensuring Lockdown

Various Nations around the world have announced lockdown across their entire country. The lockdown was released to limit the spread of COVID-19 by preventing human contact. However, there are many cases where people who are unaware of the severity of the COVID-19 infection may show adversity to some regions. Police quickly started to use drones to expand their surveillance purposes, especially in vulnerable areas around the world to make people stay inside their homes. The drones were placed separately to catch signs of lockdown violations and to help people comply with the social street rules. Countries such as Israel, China, the United States, Malaysia, Kazakhstan, Italy, France, Jordan, Belgium and Greece have deployed drones to check and collect data on blockages in public places. In Israel, drones are regularly sent to isolated areas where people are required to strictly stay at home [24], [106]. Drones are used to disperse the crowd and force people to follow social distance rules. At a meeting of the FICCI Drone Committee on April 22, 2020, one participant noted that the sharp decline in Israeli crime statistics was due to the widespread deployment of drones by the Israeli government. China has extensively deployed drones to monitor congested areas and disband crowds in cities and other regions [107]. For example, a drone network can be very useful for monitoring crowds and maintaining social distances in the cities shown in Fig. 10.

**FIGURE 9.**
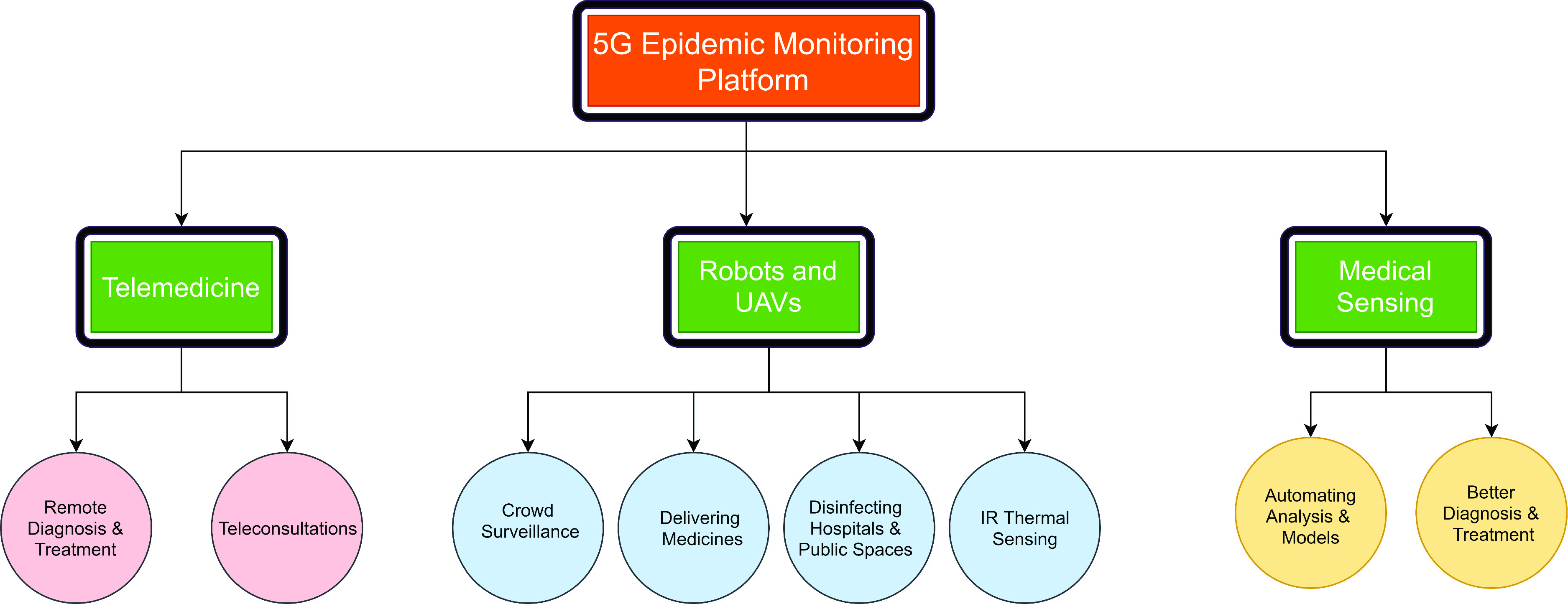
5G pandemic monitoring platform.

**FIGURE 10.**
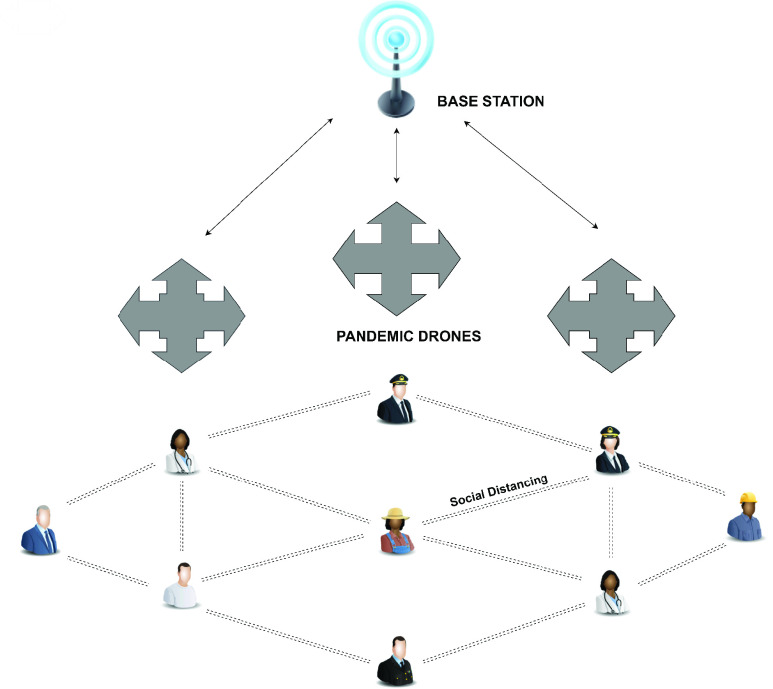
Connected drones monitoring social distancing.

##### b: Public Broadcast

Drones are positioned continuously and effectively to ensure tracking of lockdown areas, but the effect is greatly enhanced when the speaker is loaded. Drone-equipped speakers are often used to disperse crowds in public places in countries such as China, Israel, France, Spain and India [108]. Public broadcast drones not only disperse the crowd, but also publish local news related to COVID-19, which is used to educate and inform residents. In situations where some people do not wear masks [109], drone-equipped speakers encourage people to use masks as much as possible. In countries such as Malaysia, Qatar and Kuwait, speakers equipped with drones have been used to send messages in several languages [110].

##### c: Survey Mapping

Drones have been articulated to play various significant roles in activities such as survey mapping. For example, drones may perform an essential task in the study of hospitals and critical care facilities. As a matter of fact, China has explored the region using satellite technology [111]. According to this report, many abandoned areas in countries such as China, the United States and Germany have started to get converted into temporary hospitals [112]. Drones play an essential role in gathering survey maps pertaining to these areas, thus, making hospitals more efficient with less human involvement [113]. An additional drone with lights was used to illuminate the area designated for construction purposes. Drones were used to build a hospital in Wuhan, and six drones within 50 meters of the ground range were found to brighten an area of 6000 meter square and run for about 10 hours at a single charge [114].

### H. Robotics

Robotic and mechanical-based hardware visions play an important role in combating COVID-19 infection. More than one million people worldwide are infected with the COVID-19 virus. As the world continues to fight the new coronavirus pandemic, the development of robotics and machine vision technology will be an essential solution to protect people and prevent the spread of the deadly virus. These sophisticated technologies not only help in the pharmaceutical and medical device manufacturing processes but also provide treatment, identification, recognition, diagnosis and support for COVID-19 patients.

Automation should be at the forefront in order to stop the war against COVID-19. Robots should be made to treat patients in medical facilities to reduce patient-to-personnel contact. Moreover, autonomous mobile robots are coming up with different ways to keep people at a calibrated distance. In these current pandemics, the following applications are gaining much popularity.

#### 1) Vision Guided Robotics

The COVID-19 vision guide boosts much interest in robots which reduces unnecessary human contact in medical facilities and hospitals. Headquartered in the US, Arbeck is closely working with robot manufacturers in China to develop 3D camera products for robots to be used in a variety of hospital applications. Food delivery robots, aseptic robots and steering robots have been used in many hospitals in China to transport patients between hospital departments [115]

#### 2) Combating the COVID-19

##### a: Patrol Robots

China’s 5G patrol robots deployed in public places, airports and commercial complexes can monitor people’s face and their body temperatures. For example, the Guangzhou Gosungan Robot which gets powered by Advanttec’s IoT Edge Computer (MIC-770) with GPU iModule (MIC-75G20) has 5 high-resolution cameras and an infrared thermometer and can scan up to 10 temperatures simultaneously within 5 meters. If a potential danger such as a face without mask or high temperature is detected, the robot can alert the relevant authorities and initiate a real-time response.

##### b: Delivery Robot Van

Chinese startup UTI has developed a self-powered robot called Hercules for logistics applications. It has also been designed for delivering food in restricted areas. This robot is powered by lidar and stereo vision camera along with various deep learning algorithms so that it can hold up to 1,000 kg of cargo. The company plans to produce more of this autonomous logistics robot in the future [115].

#### 3) Strength

• Robots can consequently work without human impedance, which means they can attempt efficient assignments. They are helpful in conditions that are perilous for people (for example ionizing radiation or airborne illnesses).
• Besides, a robot can be normalized to guarantee predictable, blunder free execution, which isn’t influenced by tension, weariness and yearning. A few people may incline toward robot connections for specific techniques, for instance, for comfort (for example circulatory strain observing) or to keep up the security or stay away from humiliation (for example individual consideration). A robot doesn’t need protracted preparing or instructive mediations which are essential for various human specialists.

#### 4) Weakness

• Robots are costly and require numerous extensive framework to work (for example Web association, power deliverance and support). Thus, right now, this innovative technology is most appropriate to well-off for medical care associations.
• Issues with respect to contamination control presently limit the reasonableness of utilizing robots in some medical care conditions. Robots can just do assignments that they are modified to go for; thus, they are appropriate for explicit jobs and less valuable for critical thinking.
• Robots, for the most part, battle with fine engine movement which decreases their helpfulness for dexterous undertakings like cooking, dressing and opening entryways. Robots can perform monotonous undertakings for extensive stretches of time yet don’t improve with experience (except if this is essential for their programming).

##### a: Drones

Drones with specialized sensors and computer vision systems are used to identify people with infections and respiratory illnesses. It has become an essential tool for safe transportation of medical and other consumer goods, medical sample delivery and air spray disinfection [116]

##### b: Disinfectant Robots

Disinfectant robots have now started to emerge with much-advanced features. Danish security robots have been used by UVT robots in all states of China to fight the coronavirus. The self-propelled robotic operating system, when coupled with an ultraviolet mode, breaks down DNA structures and isolates diseases, viruses, bacteria, and other harmful organic microorganisms from the environment. Production of this robot has accelerated, and sales have increased in China, Asia and Europe. Similarly, Siemens and Akma started to develop a prototype of an intelligent disinfectant robot that could handle the spread of viruses [115].

In this contagious situation, robots and vision technology have much responsibility for meeting the needs of non-human labour. The demand for robotic and optical applications have surfaced in massive parts of the medical and pharmaceutical industries, but the role of robotics in intelligent manufacturing automation are indispensable solutions to mitigate the impact of today’s and future infections. Moreover, these vital automation technologies can also be used to innovate industrial production. In the present world, various manufacturers need to upgrade their facilities pertaining to automation tools and digital technology for safe and continuous operation.

## IV. Challenges, Priorities and Future Directions

**FIGURE 11.**
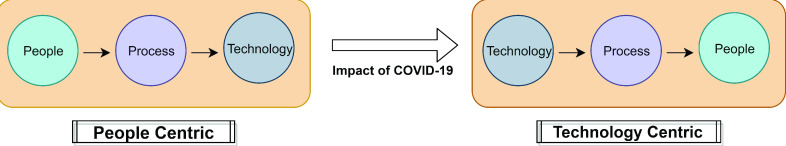
Technology-intensive architecture.

Various challenges need to be addressed especially since pandemics are generally addressed with social outreach initiatives. Development of different technologies in various areas can lead to a rapid change as seen in the history of pandemics. For instance, we can reduce exposure and limit outbreaks by making workplaces technology-intensive rather than people-intensive as shown in Fig. 11.

### A. Challenges

Variety of technical solutions faces social and ethical dilemmas, and legal and regulatory issues when applied to different sectors in the current pandemic. An exhaustive audit of developing innovative answers for COVID-19 shows that emerging technologies will employ a vital part in the crisis if the right strategies and administrative measures are set up. In the following sections, we analyze issues that arise from four key aspects: regulatory considerations, privacy and security issues, and the lack of an integrated database.

#### 1) Regulatory Consideration

The utilization of rising advancements in the clinical field to battle COVID-19 ought to be painstakingly under the current administrative structures. The capacities of different advancements can give benefits, however lawful and administrative issues can emerge if there is no responsibility to the distinctive answerable communities. For example, in a blockchain network, one needs to consider which laws and risk management controls should get applied to the system. Similarly, with respect to AI, it might be easier to build a stable framework of legal and internal governance models that direct the governance of AI applications in the healthcare industry. In particular, legal issues relating to sharing of content and personal information due to copyright, infringement and libel should also be considered [117]

#### 2) Privacy Issues

Under the application of coronavirus tracking, personal privacy is supremely paramount. The legislature can utilize mobile location data to distinguish the infection, yet this arrangement must ensure the protection of the client information, particularly privacy-based data, for example, place of residence, bank data, shopping records, etc. Government organizations might want to screen the user’s mobile usage to make different public security measures. Numerous clinical associations and organizations today are gathering information from patients with electronic clinical records to help screen manifestations and treat every illness successfully. In these medical operations, conflicts between data collection and user personal information privacy need to be resolved by strengthening laws, such that related agencies can do their work inevitably.

#### 3) Lack of Unified Databases

An essential challenge in combating corona-viruses is the lack of integrated databases such as databases for infectious diseases, infected areas, and health products. Currently, most of the corona virus-related databases come from many sources, such as social media and medical institutions. However, this is not enough for data-driven applications that want to make a significant impact in combating COVID-19. Sometimes nations are hesitant to share information leading to difficulties in understanding the outbreaks at the global level. Without access to local and global data, it will be very much challenging to develop any relevant global public health guidelines.

#### 4) Security Concerns

COVID-19 monitoring applications utilizes new technologies such as the blockchain for the sharing of personal health data. However, many shareholders are concerned about the inherent security implications of blockchain and other emerging technologies, especially with regard to the health industry records. Data threats or security attacks may target the blockchain infrastructure, which can lead to severe consequences such as viewing private medical transactions, or even worse changing patient records, exacerbating privacy concerns. Security for AI systems in medical applications is also another important are where malicious attacks can infect models by injecting false data leading to wrong diagnosis and loss of human life. Therefore, one should prioritize security issues during the design and development of technical solutions through these pandemics.

### B. Priorities

The current pandemic has distorted every dimension that defines our world. To control outbreaks and minimize the loss, our lives are governed by a bunch of needs established by the administration organizations and related policy makers which we follow judiciously.

#### 1) Flatten the Curve

The idea behind this notion is to reduce the number of critical patients so that the ratio can be maintained within the national clinical capacity. If no concrete solution is found, then aggressive social distancing (e.g., strict lockdowns), isolation and self-health are the only ways to combat this pandemic. Advances in technology continue to contribute to the development of automated approaches to help monitor these activities.

#### 2) Resilience

To stay positive in the post-COVID-19 world, one needs to guarantee the unwavering quality and the expedient recuperation of partners in the worth chain of various networks, for example, agri-business industry, social administrations and various private fields. Instead of focusing only on the economy, we need more comprehensive approach that focus on improving productivity, providing support/incentives and maintaining long-term relationships so all stakeholders in the value chain can fight together against this pandemic.

#### 3) Digital Advancements

Entering into the digital business model always requires preparing the workplaces with different technologies (e.g., AI) compatible with human creativity to meet the growing demands. These developments should focus primarily on value creation initiatives, such as non-contact work environment, automated disinfecting of work places, and various collaborative tools improving the productivity, safety and efficiency of companies.

#### 4) Handling Ecological and Environmental Threats

Severe disasters including pandemics have affected us in the past. Currently, COVID-19 is one of the most influential events, such that it adds to our concerns regarding climate change, stressed populations and global disparities. It would be helpful if the technological initiative can be future proof tackling new circumstances onward. In line with the latest trends, disruptive technologies loaded with several computer-assisted methods may mitigate the negative impact of COVID-19.

### C. Future Directions

**FIGURE 12.**
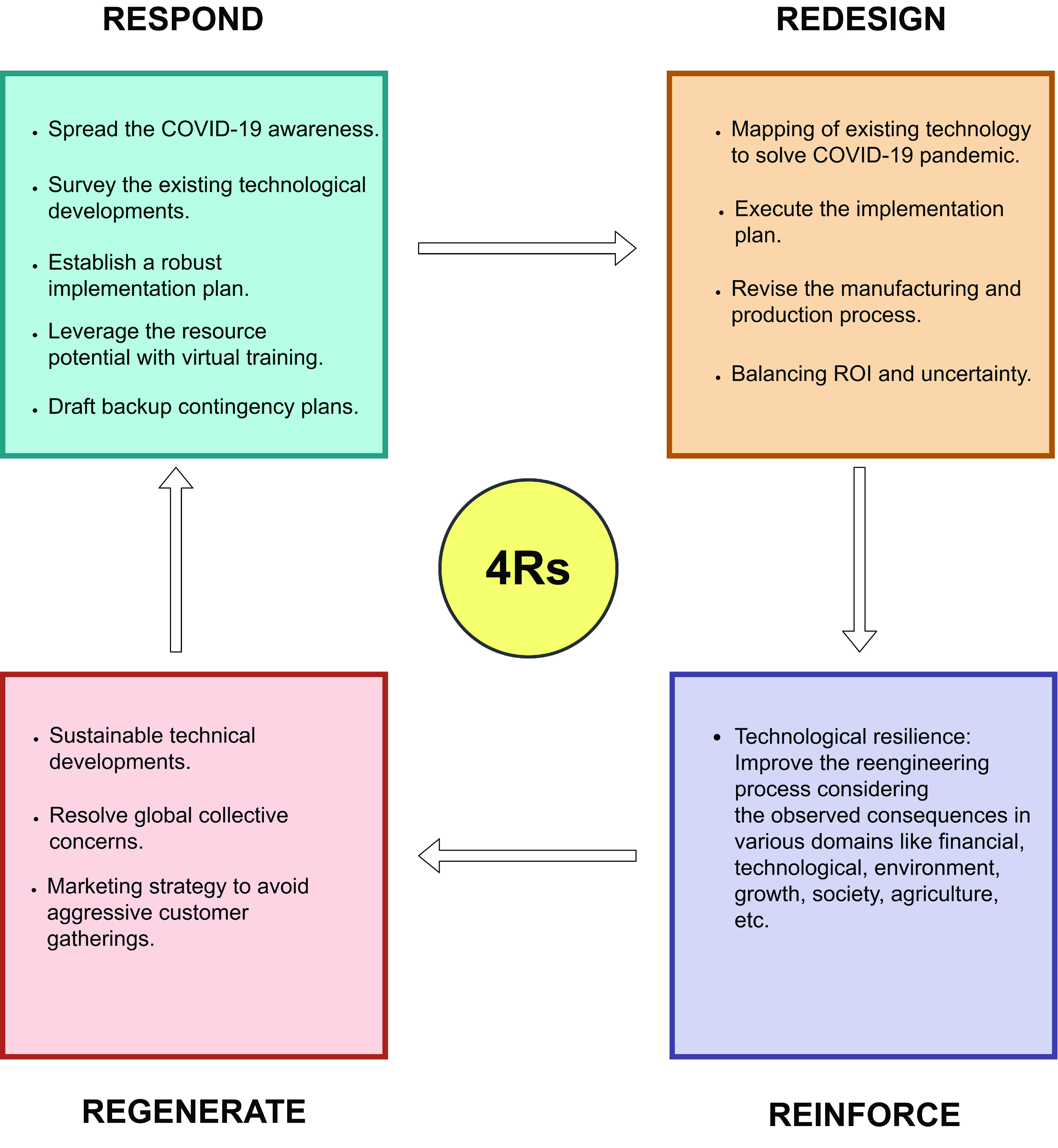
4Rs of technological action plan to combat COVID-19.

2020 has brought unprecedented challenges to the global economy. It has vastly affected companies, and countries around the world. Legal justification from policymakers and regulators of the COVID-19 pandemic situation must provide significant contributions to demonstrate the effectiveness of using emerging, and disruptive technologies. The action plan can rely on four horizons, i.e. respond, re-engineering, reinforce, and rebound as highlighted in Fig. 12. In the first phase, new technologies should be developed to fight the existing COVID-19 infections. This action is ongoing or has already been made in most countries. Once the immediate emergency is resolved through the response phase, the next step is to review the formulation of technology-based solutions in the restructuring phase and investigate serious efforts to redesign. The third phase is a reinforcement phase that involves enough time to develop technical skills based on previous results. At this point, technology can be integrated through efficient process workflow and automation to solve the COVID-19 challenge. The last phase will arise again and again until an ideal technology gets created. It is time to become conservative, moderate and aggressive so as to drive rapid and sustainable growth in the right direction for the future in all sectors. Following are future research guidelines based on technology for combating COVID-19 infection and other pandemics.

#### 1) Embracing Touchless Technology

The pandemic has set the world-leading records with many advancements in touchless technology through AI, IoT, data analysis, machine learning and more. Increased focus is made on the analysis of gestures set by human gestures, movement, voice, expression, retina, and hologram. Facial recognition for personal identities through smart cameras and sensor devices can be triggered without touching the same commands of Hologram projection-touch Assist technology.

#### 2) Optimize Blockchain for Better Deployment

To combat the COVID-19 pandemic, better healthcare systems such as patient surveillance, telemedicine and drug delivery are needed which can be realized by optimizing the blockchain platform to reduce network latency and increase efficiency, thus, improving health safety measures at various levels. More specifically, a lightweight blockchain design towards the health industry is required to enhance data verification and transactional communication for most of the delayed information transmissions.

#### 3) Accelerate AI Developments

Conventional AI approaches can also provide smart solutions to existing diseases, but they appear to less effective in an infectious disease situation. The global efforts against COVID-19 and future pandemics requires more robust and transparent techniques. With the ability to process multiple source health data, one needs to develop a specialized adaptive AI framework for clinical applications which can enhance the analysis of future intelligence data. There is also a need to utilize algorithms with more transparent outcomes in the domain of explainable AI.

#### 4) Strengthen Digital Infrastructure

Communities need to create an optimal, technical and environmental plan to strengthen the distance-work culture and reduce the communication gap between all stakeholders. It is essential to assess network-based abilities, assist gadget placement, and improve the virtual climate for a steady future. Establishing a much-conveyed workplace with strong scientific spine and improved secure network allows the crisis to move quickly with minimal loss. Investments in the cloud and communication infrastructure as well as the collaboration tools will yield positive outcomes.

#### 5) Real-Time Monitoring of IoT and Big Data Applications

Coronavirus case subtleties are accessible to all nations to direct continuous information examination to gain advanced functional insights using powerful big data tools which assists with setting up the important activity plans to safeguard numerous needs and priorities. Innovative advances such as non-contact biometrics and IoT-enabled surveillance systems have played an essential role in establishing post-COVID-19 requirements. Moreover, to achieve the goals of social distancing and closure, extensive and comprehensive data tools can be used to read measurements of the population movement, and estimate the general compliance.

## V. Conclusion

In this comprehensive review, we have presented the use of emerging and disruptive technologies to combat coronavirus (COVID-19) infection and the lessons to be learned for future pandemics. In the fight against this outbreak, this article highlighted a comprehensive list of digital technologies that have significantly improved the effectiveness of national initiatives like artificial intelligence (AI), big data, cloud computing, blockchain, 5G, and more. Infectious disease monitoring, prevention, control and treatment, and resource allocation are the critical applications that various communities should focus on shortly. Therefore, this article lays the foundation for various stakeholders to look for opportunities to turn prospects. Currently, the COVID-19 pandemic is affecting the world at all levels such that there is no successful process to stop the spread, making it impossible even to predict the finish line in our current efforts and the economic consequences. In the war against COVID-19, public privacy, data protection, and regulatory considerations are very challenging. In the interim, needs among irresistible infections have been examined to pull in the worldwide network to advance mankind. Finally, we discussed the future direction for providing the most relevant and viable research areas, scientists, and healthcare professionals. The health sector is at the forefront of combatting all infectious diseases, and the associated technologies discussed will improve performance and efficiency. However, technology cannot prevent the spread of nature-borne infections, and thus, one needs to enhance education and awareness. By the end of this review, we hope that adequate information documenting this pandemic will be generated in the form of numerous patients and drug testing reports, legal frameworks, social issues analysis, and financial studies. The use of the emerging technologies discussed by medical, social, government, economic and technological professionals are possible ways to help scientists create a digital ecosystem that monitors virus diversity and prevents population infections.
